# Repeated Multimodality Ablative Therapies for Oligorecurrent Pulmonary Metastatic Disease

**DOI:** 10.3390/curroncol29030140

**Published:** 2022-03-04

**Authors:** Alban Macagno, Alexandre de Nonneville, Pierre Annede, Gilles Piana, Isabelle Pougnet, Nassima Daidj, Laurence Moureau-Zabotto, Julien Darreon, Laetitia Padovani, Francois Bertucci, Naji Salem

**Affiliations:** 1 Department of Radiotherapy, Institute Paoli-Calmettes, CNRS, INSERM, CRCM, Aix-Marseille University, 232 Boulevard Sainte-Marguerite, 13009 Marseille, France; macagnoa@ipc.unicancer.fr (A.M.); annedep@ipc.unicancer.fr (P.A.); moureaul@ipc.uncicancer.fr (L.M.-Z.); darreaonj@ipc.uncicancer.fr (J.D.); salemn@ipc.unicancer.fr (N.S.); 2 Department of of Medical Oncology, Institute Paoli-Calmettes, CNRS, INSERM, CRCM, Aix-Marseille University, 232 Boulevard Sainte-Marguerite, 13009 Marseille, France; denonnevillea@ipc.unicancer.fr; 3 Department of Radiology, Institute Paoli-Calmettes, CNRS, INSERM, CRCM, Aix-Marseille University, 232 Boulevard Sainte-Marguerite, 13009 Marseille, France; pianag@ipc.unicancer.fr (G.P.); daidjn@ipc.unicancer.fr (N.D.); 4 Department of Radiotherapy, CRCM, La Timone Hospital, Aix-Marseille University 264 Rue Saint-Pierre, 13385 Marseille, France; isabelle.pougnet@ap-hm.fr (I.P.); Laetitia.padonvani@ap-hm.fr (L.P.)

**Keywords:** ablative therapies, oligorecurrent, oligometastasis, pulmonary metastatic disease

## Abstract

Stereotactic body radiotherapy (SBRT) and percutaneous thermal ablation (TA) are alternatives to surgery for the management of pulmonary oligometastases. In this collaborative work, we retrospectively analyzed patients who had undergone iterative focal ablative treatments of pulmonary oligometastases. We hypothesized that repeated ablative therapies could benefit patients with consecutive oligometastatic relapses. Patients treated with SBRT and/or TA for pulmonary oligometastases in two French academic centers between October 2011 and November 2016 were included. A total of 102 patients with 198 lesions were included; 45 patients (44.1%) received repeated focal treatments at the pulmonary site for an oligorecurrent disease (the “multiple courses” group). Median follow-up was 22.5 months. The 3-year overall survival rates of patients who had a single treatment sequence (the “single course” group) versus the “multiple courses” were 73.9% and 78.8%, respectively, which was not a statistically significant difference (*p* = 0.860). The 3-year systemic therapy-free survival tended to be longer in the “multiple courses” group (50.4%) than in the “single course” group (44.7%) (*p* = 0.081). Tolerance of repeated treatments was excellent with only one grade 4 toxicity. Thereby, multimodality repeated ablative therapy is effective in patients with pulmonary oligorecurrent metastases. This strategy may delay the use of more toxic systemic therapy.

## 1. Introduction

Hellman and Weichselbaum established the term “oligometastases” in 1995, for an intermediate state between loco-regional tumor spread and disseminated metastases [1,2]. This paradigm implies that patients who have an oligometastatic disease might achieve long-term survival if all detectable sites could be ablated. Although there is no precise definition of the oligometastatic state, most studies define “oligometastases” as the presence of up to three or five metastatic lesions in one or two anatomical sites [3,4,5]. Besides the metastatic burden, the timescale must be taken into consideration for defining oligometastatic state definition characterized by slow-growing kinetics. The terms “oligoprogression” and “oligorecurrence” were then coined by Palma [6]. Coincidentally, clinical data indicate that metastasis-directed ablative therapies are becoming increasingly common [7,8]. Regarding the pulmonary site, considered as one of the most frequent metastatic sites for solid tumors, percutaneous thermal-ablation (TA) therapies (radiofrequency, microwaves, and cryotherapy) and stereotactic body radiotherapy (SBRT) represent valid alternatives to the reference treatment: surgery. Although no randomized prospective trial has thus far demonstrated the benefit of these local ablative treatments compared to surgery, these procedures display different toxicity profiles and are commonly practiced with similar results in terms of local control and survival [9,10,11,12,13,14,15,16,17,18,19,20]. While not in the metastatic setting, the STARS-ROSEL trials prospectively compared surgery with SBRT for pulmonary disease (early stage/isolated nodules, though not metastases) [21,22,23,24]. In most studies, patients received only one course of ablative treatment for their metastases, either as part of the initially oligometastatic disease (synchronous oligometastases) or during an oligometastatic evolution occurring after the treatment of the primary tumor (metachronous oligometastases) [12,25,26]. However, a significant number of patients will develop a progression of metastatic disease, most often confined to the same organ [27]. Some patients then become “oligorecurrent” again, if the metastatic disease remains limited and reachable by focal treatment. To our knowledge, there are little data reported on this situation.

In this retrospective multicentric study, due to the comparable local control rates according to different treatment technics, we analyzed patients who had undergone iterative focal treatments of pulmonary oligometastases. We hypothesized that these patients, although in disease progression with additional pulmonary oligometastases, would still have potentially curable oligometastatic disease, and that repeated ablative therapies could delay more toxic systemic treatment.

## 2. Materials and Methods

### 2.1. Patient Population

We retrospectively reviewed the medical files of all consecutive patients treated between October 2011 and November 2016 by metastasis-directed ablative therapies (SBRT or TA) for lung neoplastic lesion in our two academic centers (Insitut Paoli-Calmettes and Hôpital de la Timone, Marseille, France). Patient’s data were updated until May 2018, the time of the analysis. Based on this cohort, we identified patients who had iterative focal treatments on pulmonary oligometastases. Repeated ablative treatment was defined as two or more ablative courses separated by more than three months. Lesions treated within less than three months were considered to be part of the same treatment course. Patients who had local recurrence after the first ablative treatment were included if they could have a new ablative treatment of the previously treated lesion.

### 2.2. Treatment Procedures and Follow-Up

We focused on the ablative techniques represented by SBRT and TA. When a patient had undergone pulmonary metastasis surgery before performing SBRT or TA, surgery was also considered as a therapeutic course. Regarding the SBRT procedure, we used two linear accelerators: Versa HD^®^, Elekta ™ (Insitut Paoli-Calmettes, Marseille, France) and TomoTherapy^®^, Accuray™ (Hôpital de la Timone, Marseille, France). All patients were positioned headfirst in the supine position. To provide an optimal immobilization, vacuum bags (BlueBag^®^) were used for all patients, whereas abdominal compression (BodyFix^®^) was only used for patients presenting medial or inferior tumors. A maximum intensity projection (MIP) image was performed to define the internal tumor volume (ITV). An additional planning target volume (PTV) margin of 3 mm (transversal) and 5 mm (superior-inferior) was further added to account for positioning insecurities. SBRT planning was performed with Pinnacle^®^ or RayStation^®^ treatment planning software (RaySearch Laboratories™). In March 2015, our departments of radiotherapy allowed the removal of the flattening filter to treat patients with a high dose rate beam up to 1200 MU/min for 6MV. We reported a low toxicity rate and a shortened beam time for patients treated with this procedure for lung neoplastic lesions [28]. The most commonly reported prescription doses were 48 Gy (6 fractions of 8 Gy) or 40 Gy (5 fractions of 8 Gy). In case of proximity to the organs at risk (OAR), a reduction in the dose per fraction was recommended. Daily cone-beam computed tomography was performed before each treatment session with automatic registration of the images on the tumor area.

The TA procedures included all percutaneous tumor destruction techniques with CT guidance (Siemens Somatom Definition AS64 Scanner FASTCARE, Erlangen, Germany (962252)) available in our institutions. These treatments were performed during a short hospitalization. Procedures were performed under conscious sedation or general anesthesia and were achieved according to the provisional duration of the procedure, the patients’ age, and eventual contra-indications to general anesthesia. In the case of conscious anesthesia, a cerebral target concentration of Remifantanil 2 ng/mL was used and adjusted according to the Analgesic Nociception Index. In the case of conscious sedation, the skin and underlying tissue were anesthetized using 1% Lidocaine. The CA system (Galil Medical; Yokne’am Illit, Israel) was used in all cases. Radiofrequency was the most commonly used technique. Two different types of electrodes were used: deployable electrodes (Boston Scientific^®^ LeVeen CoAccess ™ Electrode System and Boston^®^ Scientific 3000^®^ RF Generator; Marlborough, Massachusetts) and straight electrodes (Cool-Tip, 17 Gauge, and Generator Cool-Tip E series Covidien^®^). Regarding the microwave ablation technique, the generator used was an Amica generator (Ablatech^®^), associated with 14 Gauge straight needles. Cryoablation was the third technique used. The CA system (Galil Medical, Israel) was used in all cases with two different types of needles: IceRod or IceSphere. A CT scan was performed at the end of the procedure looking for complications and renewed at H24. The choice of the technique employed depended on the size and location of the lesion.

After each local ablative therapy, patients were clinically followed by a radiation oncologist or a radiologist during the treatment, then 6 weeks, 3 months, and 6 months after to detect any acute and/or subacute treatment-related toxicity. Then, long-term follow-up was performed every 6 months by a radiation oncologist or a medical oncologist, alternatively. Paraclinical exams were performed at 6 weeks, 3 months, 6 months, 1 year, and thereafter every year using 18-FDG-CT-PET or a CT-scan. The response was evaluated by either PERCIST 1.0 or RECIST 1.1 criteria for CT-PET and CT-scan, respectively.

### 2.3. Statistical Analysis

The primary endpoint was overall survival (OS). Secondary endpoints were systemic therapy-free survival (STFS), local relapse, and toxicity. Survival and local relapse were defined from the first performed local ablative procedure until the occurrence of an event or until the last monitoring consultation. Toxicities were defined by referring to the CTCAE version 4.0 (Common Terminology Criteria for Adverse Events) scale. Acute and/or subacute toxicity was considered when it occurred within 6 months of treatment.

Continuous variables were reported as median with corresponding interquartile range (IQR), whereas frequencies and proportions were used for categorical variables. Chi-squared or Student’s t-test were used to assess quantitative or qualitative variables, respectively. We performed survival analyses by using the Kaplan–Meier method to estimate OS and STFS, and survival curves were compared using the log-rank test. Univariate analyses were performed using the Cox proportional hazards model. For all analyses, a two-sided *p*-value < 0.05 was considered significant. Statistical analyses were performed using R 3.3.3 software. The study was approved by the Department of Clinical Research and Innovation of the Paoli-Calmettes Institute with the code: MULTIP-IPC 2018-044.

## 3. Results

### 3.1. Patients’ Characteristics

A total of 102 patients were included in our study corresponding to 198 treated lesions. Detailed patient characteristics are summarized in Table 1. We identified 45 patients (44.1%) who had several therapeutic sequences on the pulmonary site in an oligorecurrence context (hereafter designated “multiple courses” group), and 57 (55.9%) who had received a single therapeutic local course (hereafter designated “single course” group). There was no significant difference concerning patients’ age or baseline WHO status between the two groups. Patients with multiple sequences had significantly less significant cardiovascular history. There were differences in the distribution of primary cancers between the two groups, a colorectal or renal primary cancer being more frequent, and bronchopulmonary primary cancer being less frequent in the “multiple courses” group. There was no significant difference between the two groups regarding the number of metastatic sites initially involved, the delivery of a focal treatment for a non-pulmonary metastatic site, the time to metastasis (metachronous versus synchronous), or the delivery of a systemic treatment before ablative procedure.

### 3.2. Treatment Characteristics

Focal treatment characteristics are summarized in Table 2. The number of lesions treated by SBRT, TA, and surgery were 103, 95, and 14, respectively. Thirty-one patients had a treatment combining several types of ablative techniques either to treat several pulmonary lesions during the same sequence or during different sequences. Patients treated with SBRT had more significant central topography lesions compared to those treated with TA (47% versus 17%, respectively; *p* < 0.005). Lesions treated with SBRT were significantly larger in diameter than those treated with TA (median size: 14.5 mm versus 12 mm, respectively; *p* = 0.003).

In the “multiple courses” group, 29 patients, 12 patients, and 4 patients had received 2, 3, and 4 treatment sequences, respectively. The median interval between the first and the second, the second and the third, and the third and the fourth sequences were 13 months, 12.5 months, and 10.5 months, respectively. Pathological confirmation of secondary pulmonary metastasis was obtained in 47 patients (46.1%) at the first course of treatment. For the following sequences, pathological confirmation was only present for 5 patients (4.9%). In the “multiple courses” group, 36 patients (80%) had multiple sequences on the same lung, including 27 patients (60%) in the same lobe.

### 3.3. Patients’ Outcome

**Figure 1:**
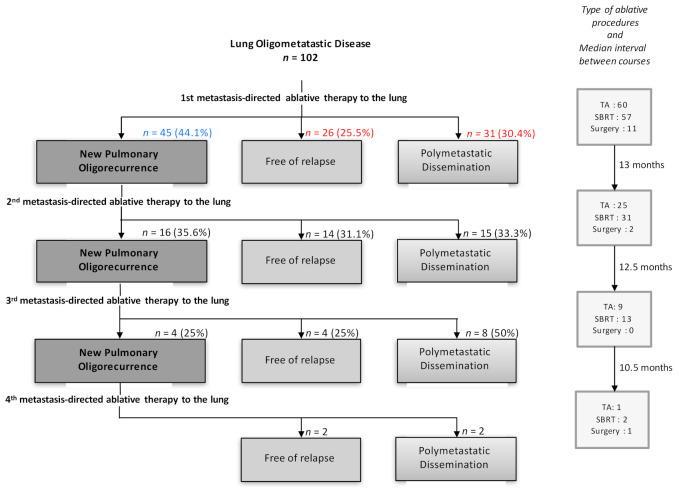
Recurrence profiles of the patients treated with multiple (in blue) or single (in red) lung metastasis-directed ablative therapies. *n*: number of patients; TA: thermo-ablation procedures; SBRT: stereotactic body radiotherapy. The colored numbers indicate the number of patients in the “single course” group (blue) and the “multiple courses” group (red).

The median follow-up of the entire cohort (102 patients) was 22.5 months (IQR 14.9–34.9). The recurrence profile of patients treated with lung metastasis-directed ablative therapies is shown in Figure 1, along with the detailed distribution in Table S1. Thirty-one out of 102 treated patients displayed multimetastatic progression after the first local ablative procedure; 26 remained free of relapse, and 45 (44%) displayed a new lung oligorecurrence that was retreated with a second, third, or fourth local ablative procedure (“multiple courses” group). Within these 45 patients, 25 displayed multimetastatic progression after one or more local ablative procedures, whereas 20 remained free of relapse. Six patients displayed a non-pulmonary oligorecurrence further treated by local procedure with curative intent.

Of the 198 lesions treated, 31 local recurrences (progression of the treated lesion) were observed: 19 lesions after initial treatment with TA, 5 of which had an efficient salvage treatment (4 SBRT and 1 TA) with no new local relapse identified; and 12 lesions after initial treatment with SBRT, 3 of which had a salvage treatment (2 TA and 1 re-irradiation) with 1 new local relapse identified.

Median OS was not reached. One-year and 3-year OS rates were 92.7% (95%CI 85.2–96.4) and 77.3% (95%CI 63.8–86.3), respectively.

**Figure 2:**
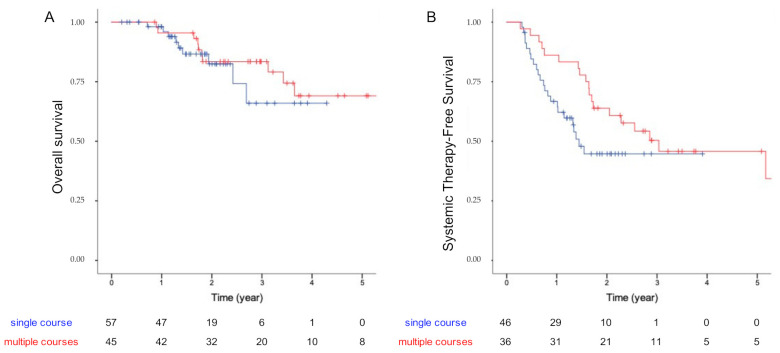
Kaplan–Meier curves showing the overall survival (**A**) and systemic therapy free survival (**B**) of the “single course” group (in blue) and “multiple courses” group (in red) treated with ablative treatment.

In univariate analysis applied to the baseline patients’ characteristics (Table 3), only the WHO status was significantly associated with OS (*p* = 0.04). Other factors tested, such as patients’ age, type of primary cancer, synchronous or metachronous evolution, numbers of non-pulmonary metastatic sites, the interval from tumor diagnosis to treatment, and prior systemic therapy, were not associated with OS, nor the number of local ablative procedures delivered to the lung lesion (“multiple courses” group versus “single course” group). Indeed, the 3-year OS rates were 73.9% (95% CI-45.3–89.1) and 78.8% (95% CI 60.8–89.2) for the “single course” group and “multiple courses” group (*p* = 0.86; Figure 2A), respectively. Similarly, in univariate analysis applied specifically to the “multiple courses” group, the type of primary cancer and interval between the first and second courses were not associated with OS.

Median STFS was 27.4 months (95% CI: 13.5–41.3). One-year and 3-year STFS were 75.3% (95% CI: 66–84.6) and 43.9% (95% CI 33.2–54.6), respectively. The 3-year STFS rate of “multiple courses” group was 50.4% (95% CI: 34.1–66.7) versus 44.7% (95% CI: 30.3–59.1) in the “single course” group (*p* = 0.081, log-rank test; Figure 2B).

**Figure 3:**
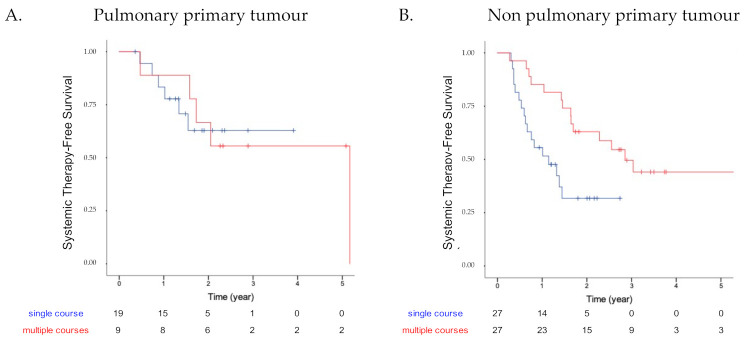
Kaplan–Meier curves showing the systemic therapy-free survival of the “single course” group (in blue) and “multiple courses” group (in red) in pulmonary primary tumor only (**A**), and in other primary localizations (**B**).

In an exploratory analysis aiming to assess STFS by primary tumor subgroups, we observed similar 3-year STFS in the “single” vs. “multiple” groups in the group of patients with pulmonary primary tumors (63% vs. 55.6%, respectively; *p* = 0.939 log-rank test; Figure 3A). By contrast, the apparent benefits of multiple courses of ablative therapy were maintained in the non-pulmonary primary tumor group with 3-year STFS of 31.7% in the “single course” group and 49.6% in the “multiple courses” group (*p* = 0.009, log-rank test; Figure 3B).

### 3.4. Toxicity

Toxicity data of the local treatments were collected for all therapeutic sequences. Grade I and II radiation-induced pneumonitis was reported in nine and seven patients treated with SBRT, respectively. Bronchial stenosis requiring endoscopic management occurred in one patient. No grade IV or V toxicity were found. For TA, a post-procedure pneumothorax was reported in 51 patients (54.8%), exsufflation was required in eight patients, and drainage also in eight. There was one grade IV toxicity requiring lobectomy after hemothorax. Residual pain at 1 and 3 months was reported in 21 patients: 16 patients experienced grade 1 pain requiring stage 1 analgesics, and five patients experienced grade ≥ 2 pain.

## 4. Discussion

Here, we retrospectively analyzed and compared the clinical data and outcomes of cancer patients who underwent iterative focal treatments for pulmonary oligometastases versus those patients who had a single course. OS was similar between the two groups of patients with 73.9% versus 78.8% (3-year OS rates in the “single course” and “multiple course” groups, respectively). Multi-treated patients were characterized by a higher proportion of metastases from colorectal and renal cancer and a lower proportion of bronchopulmonary cancer. By contrast, a difference existed concerning the 3-year STFS, which was longer in the “multiple courses” group (51.2%) than in the “single course” group (33.9%).

Few reports in the literature focused on repeated local treatments for oligometastatic disease, and our results compare favorably with those of recent studies. Klement et al. retrospectively collected data from 145 patients treated with SBRT for multiple lung metastases, including 57 who had repeated SBRT for one or more lung oligorecurrences. Most of the patients had two SBRT courses. Median OS was 23.5 months for the whole cohort and OS was not significantly influenced by the overall number of SBRT treatments. There was no additional toxicity observed in patients who had multiple SBRT at the pulmonary site [29]. Considering the pulmonary site, focal treatment choice is essential as there may be a saturation of the ablative possibilities depending on the anatomical site and the functional pulmonary reserves. Our series illustrates the fact that these focal treatments can be repeated and combined with excellent tolerance. When patients are at risk of the subsequent development of pulmonary metastases, and therefore lung function conservation is paramount, SBRT or TA may be preferred. However, when tissue confirmation is needed, surgery or TA might be more relevant for both diagnosis and local treatment. In the case of synchronous bilateral lesions, a multimodality approach could be more interesting. In addition, anatomical features must be considered in the choice of the treatment. Central lesions < 4 cm in size can be easily treated with SBRT, whereas RF is sensitive to the thermal convection of large vessels with a risk of incomplete treatment. Cryotherapy could reduce pleural irritation pain in comparison to other TA techniques. Regarding SBRT, the cumulative pulmonary dose could be a limitation for multiple courses.

One of the main findings of our study is the increased time without systemic therapy in the “multiple courses” group, especially in the non-pulmonary primary tumor group. Local treatment with ablative therapy on detectable oligometastases, reducing the tumor burden, can delay the use of systemic treatments potentially responsible for cumulative toxicities, poor quality of life, and the emergence of resistance. The patient can then claim a “systemic treatment-free interval (STFS)”. The impact of SBRT on STFS in patients affected with lung oligometastases was analyzed by Mazzola et al. The median STFS was 16 months. The oligorecurrence group had better STFS rates (*p* = 0.0035) than the lung oligoprogressive or oligopersistent disease group [30]. Merino Lara et al. described clinical outcomes following the use of extra-cranial SBRT in patients with metastatic non-small-cell lung cancer. Median OS for the 108 patients with 165 tumors was 27.3 months, and the cumulative incidence of starting/changing systemic therapy was 21.5% at 1 year after SBRT. Larger tumor size and presence of *EGFR/ALK* mutation were predictive of higher rates of starting/changing systemic therapy on multivariate analysis [31].

Our study has the limitations of all retrospective multi-center registry studies, in particular when toxicity is analyzed. However, most patients were regularly followed by a radiation oncologist, radiologist, and medical oncologist with an assessment of tolerance and effectiveness. Patient selection criteria for multiple ablative treatments were institution-specific and not standardized. Different primary histology and stages were included, but all patients had lung oligometastatic disease, and our aim was to compare the “single course” group and the “multiple courses” group that were balanced regarding several parameters associated with outcome in metastatic patients, such as the patients’ age, WHO PS, the number of metastatic sites, and the time to metastasis. Histology confirmation was performed only when there was a diagnostic doubt and/or wish to collect precious tumor samples for translational research. Surgery as a first course of local therapy may also be underestimated in our study, since we focused on TA or SBRT treatments to build our retrospective cohort (surgery was taken into account as local treatment only if performed in the selected patients). SBRT has also been shown to induce abscopal effects in several types of cancer by stimulating the systemic antitumor immune response and inducing the regression of non-irradiated metastatic lesions at a distance from the primary site of irradiation. Assessing the weight of this effect in our study cohort was unfortunately not possible since the same patient could have received a combination of SBRT, TA, and surgery during the different local treatment lines. Of note, and even if the comparison was not the point, TA toxicity was higher (58%) with pneumothorax, salvage surgery, and pain. This technique also required hospitalization (>2 days), which could impair quality of life. Patients treated with multiple ablative treatments were highly selected with a good WHO status and no significant associated comorbidity. We showed that the 3-year STFS was longer in the “multiple courses” group than in the “single course” group. This finding may reflect a selection bias (a polymetastatic relapse can occur later in the “multiple courses” group because of a better prognosis, thus delaying the systemic treatment), or the improvement of STFS could be the result of efficient local treatment on oligometastatic disease that could delay polymetastatic evolution. Additionally, high OS rates in this metastatic population should be noted. Patients included in our study were eligible to local treatment and, by definition, displayed favorable variables such as small tumor sizes (maximum: 23 mm) and a good performance status (0 to 1 in 93% of cases). Of note, more than 30% of patients who had the 1st course of ablative therapy had further polymetastatic dissemination. This could represent the suboptimal selection of patients for local treatment in real life practice in the absence of standardized and robust predictive clinical or biological markers. One of the most difficult and important issues in the treatment of oligometastatic disease is to identify those patients with a better prognosis related to the biology of the tumor itself. Oligometastatic disease remains, possibly, a completely different biological entity compared to the classic IV stage disease. Selection of patients is mandatory to offer the best treatment and to not delay systemic treatment in systemic disease. Some opponents of metastasis-directed ablative therapies will argue that the oligometastatic phenotype is intrinsically linked to the prognosis of pathology and not directly to the effect of ablative treatments. However, answers to these questions can only be considered by conducting randomized trials such as the SABR-COMET phase II trial, in which oligometastatic patients were randomized to either standard-of-care palliative chemotherapy and/or radiotherapy versus SBRT to all sites of disease [32]. In this trial, designed to detect a signal of benefit (*p* < 0.20), patients who received SBRT lived longer than those who did not. Median overall survival was 41 months for patients given SBRT, compared to 28 months in the standard treatment arm (*p* = 0.09). SBRT also doubled progression-free survival. Progression-free survival was 12 months in the SBRT arm, compared to 6 months for those who received the standard treatment (*p* = 0.001) [33].

## 5. Conclusions

Our work represents the first series of patients with repeated multimodal local treatment of pulmonary lesions for oligometastatic or oligorecurrent disease. These repeated treatments were effective with acceptable pulmonary toxicity. OS of patients treated with curative intent on several oligometastatic events was similar to those treated for a single oligometastatic event. Ablative treatments of oligometastases may delay the use of systemic treatments, with the clinician’s main concern being the maintenance of quality of life. This real-world study has the opportunity to have a significant impact, but the prospective validation of our findings, including toxicity profiles of different local therapy modalities, is required.

## Figures and Tables

**Table 1:** Patients’ characteristics.

Variable	All Patients (*n* = 102)	Single Course (*n* = 57)	Multiple Courses (*n* = 45)	*p*-Value
Age (years) median and IQR	64.3 (56.57–72.40)	64.2 (54.90–72.89)	64.1 (58.97–72.01)	0.79
Baseline WHO status				0.54
0	46 (45%)	23 (40%)	23 (51%)	
1	49 (48%)	30 (53%)	19 (42%)	
2	7 (7%)	4 (7%)	3 (7%)	
>2	0 (0%)	0 (0%)	0 (0%)	
Cardiorespiratory History	26 (25%)	20 (35%)	6 (13%)	0.02
Primary Cancer				0.03
Bronchopulmonary	36 (35%)	26 (46%)	10 (22%)	
Colorectal	18 (18%)	7 (12%)	11 (24%)	
Renal	13 (13%)	4 (7%)	9 (20%)	
Sarcoma	15 (15%)	10 (18%)	5 (11%)	
Other	20 (20%)	10 (18%)	10 (22%)	
Metastatic sites initially involved				0.19
1	71 (70%)	42 (74%)	29 (64.44%)	
2	29 (28%)	15 (26%)	14 (31.11%)	
3	2 (2%)	0 (0%)	2 (4.44%)	
Non-pulmonary focal treatment	29 (28%)	14 (25%)	15 (33%)	0.35
Brain	8 (8%)	4 (7%)	4 (9%)	
Liver	16 (16%)	8 (14%)	8 (18%)	
Other	5 (5%)	2 (4%)	3 (7%)	
Time to metastases				0.08
Synchronous	33 (32%)	23 (40%)	10 (22%)	
Metachronous	69 (68%)	34 (60%)	35 (78%)	
Systemic therapy before ablative treatment	47 (46%)	29 (51%)	18 (40%)	0.28
Chemotherapy	26 (55%)	15 (52%)	11 (61%)	
Immunotherapy	2 (4%)	2 (7%)		
Targeted therapy	5 (11%)	4 (14%)	1 (6%)	
NA	14 (30%)	8 (27%)	6 (33%)	

IQR: Interquartile range.

**Table 2:** Focal treatments characteristics.

SBRT (*n* = 103)	
Metastasis diameter (mm) (median and IQR)	14.5 (11–23)
Lung Topography	
Central	48 (47%)
Peripheral	55 (53%)
Treatment Parameters	
Dose to PTV (Gy) (median and IQR)	45.50 (40–48)
Fractionation (min–max)	6 (4–8)
BED (median and IQR)	71.25 (59.5–72)
Ipsilateral mean lung dose (Gy) (median and IQR)	6.57 (4.25–9.51)
Ipsilateral lung V20 (%) (median and IQR)	12.53 (7.25–16.96)
Ipsilateral lung V5 (%) (median and IQR)	34.58 (21.83–45.69)
TA (*n* = 95)	
Metastasis diameter (mm) (median and IQR)	12 (10–15)
Lung Topography	
Central	16 (17%)
Peripheral	79 (83%)
Techniques	
Radiofrequency	78
Microwave	4
Cryotherapy	13
Average length of hospitalization (day)	2.62
SURGERY (*n* = 14)	
Techniques	
Wedge	6
Lobectomy	5
NA	3

SBRT: Stereotactic body radiotherapy; TA: thermo-ablation procedure; IQR: interquartile range; PTV: planning target volume; BED: biological equivalent dose; VxGy: volume receiving more than x Gy.

**Table 3:** Univariate analysis for OS for whole cohort.

	Univariate Analysis		
**Factors**	**HR**	**95%-CI**	***p*-Value**
Age	1.03	0.98–1.08	0.22
WHO status > 1	3.23	1.03–10.13	0.04
Cardiorespiratory History	1.95	0.73–5.24	0.19
Number of metastatic sites initially involved	1.45	0.70–3.10	0.32
Time to metastasis (synchronous ref.)	1.31	0.49–3.50	0.59
Primary Cancer (Bronchopulmonary ref.)			
Colorectal	1.79	0.45–7.26	0.55
Renal	1.38	0.30–6.30	0.55
Sarcoma	2.85	0.76–10.69	0.55
Systemic therapy before ablative treatment	0.99	0.35–2.79	0.98
Interval from tumor diagnosis to treatment	0.99	0.86–1.15	0.96
Local relapse	0.53	0.12–2.33	0.37
Lung oligorecurrence	0.92	0.35–2.40	0.86
Multimetastatic relapse	5.81	1.33–25.28	0.0078

## Data Availability

Data are available upon reasonable request. Requests to access these datasets should be directed to corresponding author.

## Supplementary Materials

The following supporting information can be downloaded at: https://www.mdpi.com/article/10.3390/curroncol29030140/s1, Table S1: Distribution and type of locally ablative therapy according to recurrence profiles.
