# Supplementary material for: Repeated Multimodality Ablative Therapies for Oligorecurrent Pulmonary Metastatic Disease
Source: Curr Oncol. 2022 Mar 4;29(3):1683–94. doi: 10.3390/curroncol29030140 (PMC8947282; doi:10.3390/curroncol29030140)
Supplement: Supplementary file 1 [file curroncol-29-00140-s001.zip › curroncol-1516442-supplementary.pdf]

Supplementary Material

Repeated Multimodality Ablative Therapies for Oligorecurrent Pulmonary Metastatic Disease

Alban Macagno [1], Alexandre de Nonneville [2], Pierre Annede [1], Gilles Piana [3], Isabelle Pougnet [4], Nassima Daidj [3], Laurence Moureau-Zabotto [1], Julien Darreon [1], Laetitia Padovani [4], Francois Bertucci [2,*] and Naji Salem [1]

[1] Department of Radiotherapy, Institute Paoli-Calmettes, CNRS, INSERM, CRCM, Aix-Marseille University, 232 Boulevard Sainte-Marguerite, 13009 Marseille, France ; macagnoa@ipc.unicancer.fr (A.M.); annedep@ipc.unicancer.fr (P.A.); moureaul@ipc.uncicancer.fr (L.M.-Z.); darreaonj@ipc.uncicancer.fr (J.D.); salemn@ipc.unicancer.fr (N.S.)
[2] Department of of Medical Oncology, Institute Paoli-Calmettes, CNRS, INSERM, CRCM, Aix-Marseille University, 232 Boulevard Sainte-Marguerite, 13009 Marseille, France; denonnevillea@ipc.unicancer.fr
[3] Department of Radiology, Institute Paoli-Calmettes, CNRS, INSERM, CRCM, Aix-Marseille University, 232 Boulevard Sainte-Marguerite, 13009 Marseille, France; pianag@ipc.unicancer.fr (G.P.); daidjn@ipc.unicancer.fr (N.D.)
[4] Department of Radiotherapy, CRCM, La Timone Hospital, Aix-Marseille University 264 Rue Saint-Pierre, 13385 Marseille, France; isabelle.pougnet@ap-hm.fr (I.P.); Laetitia.padonvani@ap-hm.fr (L.P.)
* Correspondence: bertuccif@ipc.unicancer.fr; Tel.: +33-4-91223537

Table S1. Distribution and type of locally ablative therapy according to recurrence profiles.

	**Patients (*n*)**	**TA (*n*)**	**SBRT (*n*)**	**Surgery (*n*)**
**First metastasis-directed ablative therapy to the lung**	102	60	57	11
Free of relapse	26*	14	16	0
Polymetastatic dissemination	31*	16	15	0
New pulmonary oligorecurrence	45*	30	26	11
**Second metastasis-directed ablative therapy to the lung**	45	25	31	2
Free of relapse	14	5	13	1
Polymetastatic dissemination	15	6	9	1
New pulmonary oligorecurrence	16	14	9	0
**Third metastasis-directed ablative therapy to the lung**	16	9	13	0
Free of relapse	4	3	2	0
Polymetastatic dissemination	8	3	5	0
New pulmonary oligorecurrence	4	3	6	0
**Fourth metastasis-directed ablative therapy to the lung**	4	1	2	1
Free of relapse	2	0	1	1
Polymetastatic dissemination	2	1	1	0
New pulmonary oligorecurrence	0	0	0	0

*, the figures in red represent the 57 patients of the “single course“ group and in blue the 45 patients of the “ multiple courses“ group.
